# Monetary Reward Discounting, Inhibitory Control, and Trait Impulsivity in Young Adults With Internet Gaming Disorder and Nicotine Dependence

**DOI:** 10.3389/fpsyt.2021.628933

**Published:** 2021-01-28

**Authors:** Wan-Sen Yan, Ruo-Ting Chen, Meng-Meng Liu, Dan-Hui Zheng

**Affiliations:** Department of Psychology, School of Medical Humanitarians, Guizhou Medical University, Guiyang, China

**Keywords:** Internet Gaming Disorder, Nicotine Dependence, inhibitory control, reward discounting, impulsivity

## Abstract

Internet Gaming Disorder (IGD) has been considered a potential behavioral or non-substance addiction that requires further investigation. Recognition of the commonalities between IGD and Substance Use disorders (SUD) would be of great help to better understand the basic mechanisms of addictive behaviors and excessive Internet gaming. However, little research has targeted a straightforward contrast between IGD and SUD on neuropsychological aspects. The present study thus aimed to explore the associations of reward processing and inhibitory control with IGD and nicotine dependence (ND) in young adults. Fifty-eight IGD and 53 ND individuals, as well as 57 age- and gender-matched healthy controls, were assessed with a series of measurements including the Delay-discounting Test (DDT), Probability Discounting Test (PDT), the Stroop Color-Word Task, a revised Go/No Go Task, and the Barratt Impulsiveness Scale (BIS-11). Multivariate analysis of variance (mANOVA) models revealed that both IGD and ND groups scored higher than healthy controls on the BIS-11 attentional, motor, and non-planning impulsiveness (Cohen's *d* = 0.41–1.75). Higher degrees of delay discounting on the DDT were also found in IGD and ND groups compared to healthy controls (Cohen's *d* = 0.53–0.69). Although IGD group did not differ from healthy controls on the PDT, ND group had a lower degree of probability discounting than healthy controls (Cohen's *d* = 0.55), suggesting a reduction in risk aversion. Furthermore, ND subjects showed a lower correct accuracy in the incongruent trials of the Stroop task than healthy controls (Cohen's *d* = 0.61). On the Go/No Go task, both IGD and ND groups had a lower correct accuracy in the No-Go trials than healthy controls (Cohen's *d* = 1.35–1.50), indicating compromised response inhibition. These findings suggested that IGD was linked to both anomalous reward discounting and dysfunctional inhibitory control, which was comparable with one typical SUD category (i.e., ND). This study might promote a better understanding of the pathogenesis of IGD as a potential addictive disorder similar to SUD.

## Introduction

Internet Gaming Disorder (IGD) has been included as a tentative behavioral or non-substance addiction that warrants further research before it can be accepted as a full disorder in the latest revision of the Diagnostic and Statistical Manual of Mental Disorders (i.e., DSM-5) (1). More recently, IGD was proposed in the list of addictive conditions and was formally recognized as Gaming Disorder in the 11th revision of the International Classification of Diseases (i.e., ICD-11) (2–4). Both in the DSM-5 and ICD-11, IGD is characterized by a pattern of persistent and disordered gaming behavior, which leads to significant clinical impairments within a period of at least 12 months (5, 6). To be diagnosed as IGD in the DSM-5, five of the nine diagnosis criteria (i.e., preoccupation, withdrawal, tolerance, loss of control, loss of interest or giving up other activities, continued overuse, deception, escape of negative feelings, negative consequences) must be endorsed within a 12-month period (1). Prevalence estimates of IGD among general samples have been always below 5% (7, 8), with a low of 0.5% and a high of 10% (9). In recent meta-analysis studies, the global prevalence of IGD was reported to be about 3.05% with significant variability (10), ranging from 0.21 to 57.5% in general populations, 3.2–91.0% in clinical populations, and 50.42–79.25% in populations undergoing intervention (i.e., severe cases) (11).

As a putative non-substance addiction, IGD has led to a large number of issues, concerns, and scientific dialogues from experts in the field (12–16). Although IGD seems to share many clinical manifestations with Substance Use disorders (SUD) in terms of etiology, biology, and treatment (13, 17–19), it remains a highly controversial topic whether IGD should qualify as a new clinical disorder (12, 20, 21), and a wider range of empirical studies are needed to clarify the theoretical underpinnings of IGD (2). In a manner, understanding the biological, psychological, and social processes underlying different forms of addictive behaviors stands to capture the core elements of IGD, such as on the commonalities and distinctions between IGD and SUD (22).

However, little research by now has targeted a straightforward contrast between IGD and other well-identified addictions on neuropsychological aspects. Considering the core features of impulsivity and compulsivity involved in addictive behaviors, one prior study has tried to detect the similarities and differences among male patients with IGD, Gambling Disorder (GD), and Alcohol Use Disorder (AUD) compared to healthy controls with a small sample size (23). It was reported that the IGD and AUD groups had higher impulsivity scores on the Barratt Impulsiveness Scale (BIS-11) and showed decreased proportions of successful stops on the Stop-Signal Test than the healthy controls, while only the GD group made more errors on the compulsivity test (i.e., the Intra-Extra Dimensional Set Shift Test) compared with healthy controls (23). Another latest study assessed trait impulsivity, delay discounting, and decision making between patients with IGD and GD compared to healthy controls, reporting that IGD and GD groups did not differ from healthy controls on the BIS-11, but both groups displayed a steeper delay curve (i.e., a higher discounting degree) on the Delay Discounting Task (DDT) (24). Despite these preliminary evidence, recognition of the commonalities between IGD and SUD/GD would be of great help to better understand the basic mechanisms of IGD from a cross-spectrum view (25).

Relative to other populations, adolescents and young adults have been found to be more susceptible to IGD because of their age-related immaturity of cognitive control as well as their easy access to the Internet during this period (26–31). Analogously, cigarette smoking behavior (or even worse, Nicotine Dependence) as one kind of SUD categories has also been available and increasing in youths from middle schools to universities (32–35), sometimes equally between males and females (36). In many cases, Nicotine Dependence (ND) and IGD tend to co-occur in young men (37, 38), and there is a high co-occurrence of cigarette smoking with IGD in both adults and adolescents (39). Interestingly, although significant correlations of IGD with various forms of SUD including nicotine, alcohol, caffeine, and cannabis use were found in the adult and elder populations (39), cigarette smoking, rather than other substance use, was strongly associated with IGD in the adolescent and younger populations (40). Moreover, cue-induced smoking craving and gaming urge showed similar neurobiological correlates (e.g., higher parahippocampus activation) in young adult subjects comorbid with ND and IGD (37), and young ND and IGD individuals shared decreased resting-state functional connectivity of the dorsolateral prefrontal cortex with the right insula and left inferior frontal gyrus, which are related to craving and impulsive inhibitions (41). Nevertheless, the common and distinct aspects of neuropsychological characteristics between IGD and ND are not well-acknowledged given the scarce evidence with a direct comparison between them.

According to recent neurocognitive models of addiction, the neural substrates implicated in addictive behaviors might include multiple brain systems that govern reward seeking/risk taking, craving and cognitive control (42, 43). Indeed, individuals with IGD are often characterized by heightened reward-seeking, persistent craving, and decreased executive control (44–46). Furthermore, the developmental theories of adolescent brains highlighted the imbalance between a salient reward-seeking system and a hypoactive executive-control system, which is closely associated with various risky behaviors including IGD during adolescence and early adulthood (47, 48). Thus, it is necessary to extend the neurocognitive underpinnings of IGD by evaluating both reward processing and cognitive control among adolescents and young adults with IGD, especially in direct contrast to those with other addictive behaviors (e.g., ND).

The Delay-discounting Test (DDT) (49) is a widely-used reward choice task that assesses the ability of delay of gratification by choosing between immediate and prospective monetary rewards (50). Similarly, the Probability Discounting Test (PDT) (51) evaluates the propensity of taking a risk for gaining more valuable rewards by choosing between one smaller reward delivered “for sure” and another larger but probabilistic reward (52). Previous case-control studies have consistently revealed a higher degree of delay discounting among adolescents and young adults with IGD (53–58), though some data showed no differences between problematic and normal Internet users on the DDT (25). More interestingly, treatment seekers diagnosed with IGD displayed a similar tendency on discounting long-term rewards faster with those diagnosed with gambling disorder (24). In heavy smokers and nicotine-dependent individuals, greater delay-discounting rates were also found (59–63), and the degree of delay discounting was significantly related to the severity of ND (64, 65). However, regarding probability discounting, limited data have been discrepant. Some studies showed that IGD participants preferred the probabilistic rewards to those delivered “for sure,” compared to recreational Internet game users and healthy controls (66–68). Nonetheless, some adolescents and college students with IGD revealed no differences on the PDT compared to healthy controls (53, 54). Analogously, some data revealed that heavy smokers showed a shallower rate of probability discounting than never- smokers (63, 68), while more studies displayed no differences between heavy/habitual smokers and never-smokers on the PDT (60, 69–71), and acute smoking abstinence did not reveal an increase in probability discounting of money or cigarettes (72).

Together with reward processing, cognitive control is believed to play a critical role in the transition from recreational drug use to drug addiction, given the fact that some individuals who use addictive drugs finally develop an addiction while others do not (73–77). Abnormalities in cognitive control have been observed in IGD samples (67, 78, 79), accompanied by neural alterations in the prefrontal regions (18, 80, 81). Although cognitive control consists of a series of cognitive processes that regulate goal-directed actions and adaptive responses to complex situations, such as response inhibition, performance monitoring, and working memory (82), most studies concerning IGD mainly investigated inhibitory control or response inhibition (83, 84). Previous studies revealed that adolescents with IGD committed more errors in the incongruent conditions than healthy controls on the Stroop tasks (85–88). Adolescents and young adults with IGD also made more commission errors in no-go trials on the Go/No-Go tasks, and showed longer stop-signal reaction time (SSRT) on the Stop- Signal tasks (23, 31, 89, 90). Nevertheless, some studies did not reveal differences on the Go/No-Go task between IGD and healthy control groups (91–93). Except for the diverse samples, these inconsistent results might also be due to the mixed processing of both stimulus-driven attentional bias and response inhibition in the Go/No-Go task itself (i.e., novelty from 25% No-Go trials vs. 75% Go trials). A newly modified Go/No-Go task, containing 75% frequent-Go trials, 12.5% infrequent-Go trials, and 12.5% No-Go trials, has been developed to directly detect response inhibition (94). A clear association between inhibitory control taxed by this novel task and smoking relapse vulnerability was revealed in treatment-seeking smokers (95). Nonetheless, deficits in inhibitory control were not consistently found in ND. Some data revealed that inhibitory control performance was negatively correlated with smoking behavior (96, 97), and subjects with ND showed impaired inhibitory control following 12-h abstinence (98). However, heavy smokers and non-smokers displayed no differences on the classical Go/No-Go tasks (99, 100), though smokers committed more errors on the Stroop task (101). Given the mixed tasks used in the literature and no direct comparison between IGD and ND, it remains unclear whether inhibitory control dysfunctions are simultaneously connected to IGD and ND in young adults.

Therefore, the main purpose of this current study was to gather more empirical evidence about the associations of reward processing and inhibitory control with both IGD and ND among young adults, targeting a straightforward contrast between IGD individuals, ND individuals, and healthy controls on the Delay-discounting Test (DDT), the Probability Discounting Test (PDT), the Stroop Color-Word Task, and the revised Go/No Go Task. Besides, we also employed the Barratt Impulsiveness Scale (BIS-11) to test trait impulsivity, considering the inconsistent BIS-11 data between IGD and other addictive disorders (23, 24). We generally hypothesized that as a putative non-substance addiction, IGD might share an aberrant pattern of inhibitory control and reward discounting with ND that is one typical kind of SUD categories.

## Methods

### Participants and Procedure

A total of 168 young adult subjects participated in this study, including 58 individuals with Internet Gaming Disorder (IGD; mean age: 20.19 ± 1.42 years; 35 males, 60.3%), 53 individuals with Nicotine Dependence (ND; mean age: 20.64 ± 1.72 years; 33 males, 62.3%), and 57 age- and gender-matched healthy controls (HC; mean age: 20.19 ± 1.41 years; 36 males, 63.2%). All of them were college students recruited during April and September 2019, through advertisement and flier from two local universities in Guiyang City, China. Participants were invited to complete a person-to-person screening interview conducted by an experienced psychiatrist and a well-trained clinical psychologist in the laboratory, and then they finished a battery of questionnaires and cognitive tasks when enrolled according to the clinical interview.

Inclusion criteria for the IGD group included: (1) ≥18 years of age; (2) meeting five or more of the nine criteria for IGD proposed in the DSM-5 (1); (3) having a score of 50 or more on the Internet Addiction Test (IAT) (102), which indicates severe or problematic Internet use (103); and (4) at least 3 h per day spent on playing Internet games (mainly the Multiplayer Online Battle Arena games, such as the League of Legends, the Arena of Valor, and the Game For Peace/Playerunknown's Battlegrounds) over a 12-month period. The exclusion criteria included current/past major psychiatric disorders (e.g., schizophrenia, bipolar disorder), neurological diseases or mental disorders, brain trauma, use of psychoactive drugs (e.g., cocaine, heroin, methamphetamine), alcohol abuse or dependence, and current/past smoking.

Inclusion criteria for the ND group included: (1) ≥18 years of age; (2) endorsing three or more of the seven criteria for Nicotine Dependence in the DSM-IV-TR (104); (3) having a score of 4 or more on the Fagerström Test for Nicotine Dependence (FTND) (105), which is determined as high nicotine dependence (106, 107); and (4) daily smoking with at least 10 cigarettes over a 12-month period. Moreover, the ND group should have no history of regular Internet gaming, with a score of <40 on the IAT indicating normal Internet use (103). The exclusion criteria were same as those for the IGD group (except for the criterion of current/past smoking).

The healthy controls met the following criteria: (1) ≥18 years of age; (2) a score of <40 on the IAT and no experience of Internet gaming; (3) non-smoking and a score of 0 on the FTND; and (4) no current/past major psychiatric disorders, neurological diseases or mental disorders, brain trauma, use of psychoactive drugs, alcohol abuse or dependence. All subjects were right-handed and had normal or rectified eyesight, without any color vision deficiency. All of them gave written informed consent and were compensated with a gift equal to RMB ¥ 50 for their time. The current study was reviewed and approved by the Human Research Ethics Committee at the Guizhou Medical University. The proposed study design, recruitment process, and our plans to compensate the participants were in accordance with the Declaration of Helsinki.

### Monetary Reward Discounting Tasks

We used the Delay-discounting Test (DDT) (49) and Probability Discounting Test (PDT) (51) to assess discounting degrees of rewards in the context of monetary choice. The DDT contains a set of choices between a smaller immediate reward and a larger delayed reward. The degree of delay discounting is calculated by the hyperbolic equation *V* = *A*/(1+*kD*). In this equation, *k* is a free parameter, with a larger *k*-value describing a higher degree of delay discounting. An adapted version of DDT among Chinese students (108) was used in this study, as reported in our previous studies (25, 109). Examples of choices on this task are “A: receiving RMB ¥1000 now; B: receiving RMB ¥10000 one year later” and “A: receiving RMB ¥9000 now; B: receiving RMB ¥10000 one year later.” The *k-*value was calculated and log-transformed in keeping with the literature. The PDT consists of three parts (i.e., Part A: $20 vs. $80; Part B: $40 vs. $100; Part C: $40 vs. $60), with 10 choices in each part. Subjects have to choose between a smaller amount of money delivered “for sure” and a larger amount of money delivered probabilistically. Examples of choices are “A: $20 for sure; B: a 1-in-10 chance/10% of winning $80” and “A: $40 for sure; B: a 5-in-10 chance/50% of winning $100.” This task has been properly used in our previous study reported elsewhere (109). The degree of probability discounting is calculated by the equation *V* = *A*/(1+*h*Θ), in which the free parameter *h* refers to the degree of probability discounting. Lower *h* indicates that the probabilistic rewards are less steeply discounted, thus suggesting a reduction in risk aversion or a higher level of risk-taking (51). The *h* scores in each part were calculated and log-transformed to get a normal distribution as suggested before.

### Inhibitory Control Tasks

The standard Stroop Color-Word Task (110) was used to measure respond inhibition under cognitive interference condition. In this task, participants are instructed to name the color of the words that are printed in a certain ink. There are two kinds of trials. In congruent trials, the word is printed in a concordant color (e.g., the word RED printed in red ink), while in the incongruent trials, the word-color pairs are always conflicting (e.g., the word RED printed in green ink). In our study, the colored words were presented on the black screen in a 7 × 7 cm size. Each word was presented for 1350ms, with a total interstimulus interval of 2000ms, according to previous studies (111). There were 54 word-color pairs each in the congruent and incongruent trials, and the task lasted for about 6 min. Subjects had 8 trails to check up on the response keys (e.g., “1” for RED, “2” for GREEN) before formal experiments. This task was programmed using the E-prime Version 2.0 (Psychology Software Tools, Inc., Sharpsburg, PA, USA). Response time (RT) and correct accuracy in the congruent and incongruent trials were analyzed.

A modified and validated Go/No Go Task (94, 95) was employed to investigate inhibitory control or response inhibition. This task was designed to separate the processing of infrequent stimuli (e.g., stimulus-driven attention) from inhibitory processes by including three different types of colored circles: frequent-go trials (frequent gray, *n* = 388, about 75%), infrequent-go trials (rare yellow, *n* = 65, about 12.5%), and no-go trials (rare blue, *n* = 65, about 12.5%). The contrast of the no-go trials vs. the infrequent-go trials was expected to detect the process of response inhibition. In this task, a colored circle was presented on the black screen for 400 ms with a 400-ms interstimulus interval over 7 min. Participants were told to press a button as quickly as possible with the right index finger in response to gray and yellow circles, but to withhold a response to blue circles. The frequent-go, infrequent-go, and no-go trials were intermixed in pseudo-random order. Prior to the formal experiments, subjects practiced 30 filler trials (10 gray, 10 yellow, and 10 blue circles). This task was also programmed using the E-prime Version 2.0. Reported no-go accuracy was adjusted, including just those no-go trials with a correct response to the preceding go trial, to control for the effects of attentional lapses (94, 95).

### Trait Impulsivity Measurement

The Barratt Impulsiveness Scale-11 (BIS-11) (112) was employed to measure impulsive traits on three dimensions (Motor Impulsiveness, Attentional Impulsiveness, Non-planning Impulsiveness). Each dimension consists of 8 or 11 items that are rated on a 4-point scale (1 = rarely/never, 4 = almost always/always). Scores of each dimension were obtained for analyses, with higher scores indicating higher levels of trait impulsivity. Cronbach's α was 0.69–0.81 for the three dimensions in this study.

### Data Analysis

All data were analyzed with the Statistical Package for the Social Sciences for Windows, Version 19.0 (SPSS Inc., Chicago, IL, USA). Categorical data such as gender, ethnicity, and home locality were analyzed with chi-square tests for group comparisons. The 3 (group: IGD, ND, HC) × 2 (gender: male, female) multivariate analysis of variance (mANOVA) models were used to compare task scores. *Post-hoc* tests were conducted using Fisher's least significant differences protected *t*-tests. Partial correlations were tested between task performance and gaming/smoking variables in IGD and ND groups, controlling for gender, age, ethnicity, and home locality. Statistical significance was set as *p* < 0.05, two-tailed.

## Results

### Demographic Characteristics and Trait Impulsivity

Table 1 describes the demographic characteristics and BIS-11 scores of the Internet Gaming Disorder (IGD), Nicotine Dependence (ND), and healthy controls (HC) groups. No between-group differences were detected on age [*F*_(2, 165)_=1.597, *p* = 0.260], gender (χ^2^ = 0.101, *p* = 0.951), ethnicity (χ^2^ = 0.211, *p* = 0.900), or home locality (χ^2^ = 0.090, *p* = 0.956). IGD group had a higher IAT score than ND and HC groups [*F*_(2, 165)_= 752.96, *p* < 0.001]. On the BIS-11, the 3 (group: IGD, ND, HC) × 2 (gender: male, female) mANOVA model revealed significant between-group effects on all of the three dimensions, including Motor Impulsiveness [*F*_(2, 162)_ = 8.255, *p* < 0.001, $\eta_{\text{p}}^{2}=$ 0.092], Attentional Impulsiveness [*F*_(2, 162)_ = 44.111, *p* < 0.001, $\eta_{\text{p}}^{2}=$0.353], and Non-planning Impulsiveness [*F*_(2, 162)_= 5.867, *p* = 0.003, $\eta_{\text{p}}^{2}$ = 0.068]. Pairwise comparisons showed that both IGD and ND groups scored higher than healthy controls on Motor Impulsiveness (Cohen's *d* = 0.78, *p* < 0.001; Cohen's *d* = 0.53, *p* = 0.007, respectively), Attentional Impulsiveness (Cohen's *d* = 1.75, *p* < 0.001; Cohen's *d* = 0.92, *p* < 0.001, respectively), and Non-planning Impulsiveness (Cohen's *d* = 0.64, *p* = 0.001; Cohen's *d* = 0.41, *p* = 0.049, respectively). The IGD and ND groups did not differ from each other on Motor Impulsiveness (*p* = 0.253) or Non-planning Impulsiveness (*p* = 0.172), but IGD group scored higher than ND group on Attentional Impulsiveness (Cohen's *d* = 0.83, *p* < 0.001). Main effects of gender and interaction effects of group × gender were not significant on any of these dimensions (*ps* > 0.05).

**Table 1:** Demographic characteristics and BIS-11 scores for the three groups.

**Variables**	**a. IGD (*n* = 58)**	**b. ND (*n* = 53)**	**c. HC (*n* = 57)**	***F*/χ^2^**	***p***	***Post-hoc* test (*p* < 0.05)**
Age, years (*M ± SD*)	20.19 ± 1.42	20.64 ± 1.72	20.19 ± 1.41	1.597	0.206	-
Gender, Male *n* (%)	35 (60.3)	33 (62.3)	36 (63.2)	0.101	0.951	-
Ethnicity, Hans *n* (%)	42 (72.4)	37 (69.8)	42 (73.7)	0.211	0.900	-
Home locality, Urban *n* (%)	34 (58.6)	31 (58.5)	32 (56.1)	0.090	0.956	-
IAT score (*M ± SD*)	67.83 ± 7.67	33.06 ± 3.88	31.88 ± 4.40	752.96^***^	<0.001	a>b, a>c
Years of regular gaming (*M ± SD*)	3.91 ± 1.34	-	-	-	-	-
Daily gaming hours (*M ± SD*)	5.19 ± 1.92	-	-	-	-	-
FTND score (*M ± SD*)	0.00 ± 0.00	5.83 ± 1.03	0.00 ± 0.00	-	-	-
Years of smoking (*M ± SD*)	-	4.89 ± 1.63	-	-	-	-
Cigarettes per day (*M ± SD*)	-	15.08 ± 6.34	-	-	-	-
**BIS-11 SCORE (*****M*** **±*****SD*****)**						
Motor impulsiveness	21.88 ± 3.81	20.87 ± 3.36	19.14 ± 3.20	9.134^***^	<0.001	a>c, b>c
Attentional impulsiveness	20.36 ± 3.32	17.68 ± 3.16	14.86 ± 2.97	43.874^***^	<0.001	a>b>c
Non-planning impulsiveness	29.86 ± 4.56	28.66 ± 3.43	27.14 ± 3.93	6.625^**^	0.002	a>c, b>c

*IGD, Internet Gaming Disorder; ND, Nicotine Dependence; HC, Healthy Controls; IAT, Internet Addiction Test; FTND, Fagerström Test for Nicotine Dependence; BIS, Barratt Impulsiveness Scale*.

** *p < 0.01*,

*** *p < 0.001*.

### Monetary Reward Discounting

The scores on the delay-discounting and probability-discounting tasks of the IGD, ND, and HC groups are displayed in Table 2. The mANOVA models showed that group effects were significant on the DDT score (i.e., log-transformed *k* value) and on the PDT score (i.e., log-transformed *h* value) of the Part A [*F*_(2, 162)_ = 7.505, *p* = 0.001, $\eta_{\text{p}}^{2}$ = 0.085; *F*_(2, 162)_ = 7.118, *p* = 0.001, $\eta_{\text{p}}^{2}$ = 0.081, respectively], but not on the PDT Part B or Part C scores [*F*_(2, 162)_ = 2.975, *p* = 0.054; *F*_(2, 162)_ = 2.674, *p* = 0.072, respectively]. Pairwise comparisons revealed that both IGD and ND groups had a higher degree of delay discounting (i.e., log-transformed *k-*value) on the DDT than healthy controls (Cohen's *d* = 0.53, *p* = 0.002; Cohen's *d* = 0.69, *p* = 0.001, respectively), but the difference between IGD and ND groups was not significant (*p* = 0.253). By contrast, ND group had a lower probability-discounting degree (i.e., log-transformed *h-*value) on the PDT (Part A), compared with healthy controls (Cohen's *d* = 0.55, *p* = 0.004) and IGD group (Cohen's *d* = 0.79, *p* < 0.001), but IGD group did not differ from healthy controls (*p* = 0.546). Main effects of gender and interaction effects of group × gender were not significant on any of the DDT and PDT scores (*ps* > 0.05).

**Table 2:** Discounting degrees of the three groups on the DDT and PDT.

**Variables**	**a. IGD (*n* = 58)**	**b. ND (*n* = 53)**	**c. HC (*n* = 57)**	***F***	***p***	***Post-hoc* test (*p* < 0.05)**
**DDT score (*****M*** **±*****SD*****)**						
*k* value	0.34 ± 0.22	0.35 ± 0.19	0.25 ± 0.22	3.626^*^	0.029	a>c, b>c
*k* value (log-transformed)	−0.60 ± 0.41	−0.55 ± 0.32	−0.85 ± 0.53	7.571^**^	0.001	a>c, b>c
**PDT score (*****M*** **±*****SD*****)**						
*Part A ($20 vs. $80):*						
*h* value	5.61 ± 4.39	3.51 ± 4.02	6.26 ± 4.67	5.870^**^	0.003	a>b, c>b
*h* value (log-transformed)	0.62 ± 0.35	0.28 ± 0.50	0.57 ± 0.56	8.009^***^	<0.001	a>b, c>b
*Part B ($40 vs. $100):*						
*h* value	3.66 ± 4.53	2.41 ± 3.09	4.12 ± 3.98	2.773	0.065	-
*h* value (log-transformed)	0.31 ± 0.45	0.14 ± 0.46	0.37 ± 0.52	3.596^*^	0.030	c>b
*Part C ($40 vs. $60):*						
*h* value	3.35 ± 4.99	1.89 ± 3.32	3.06 ± 4.78	1.643	0.197	-
*h* value (log-transformed)	0.14 ± 0.54	0.06 ± 0.48	0.16 ± 0.48	3.383^*^	0.036	c>b

*IGD, Internet Gaming Disorder; ND, Nicotine Dependence; HC, Healthy Controls; DDT, Delay-discounting Test; PDT, Probability Discounting Test; k represents the delay-discounting degree, h represents the probability-discounting degree*.

* *p < 0.05*,

** *p < 0.01*,

*** *p < 0.001*.

### Inhibitory Control Performance

**Figure 1:**
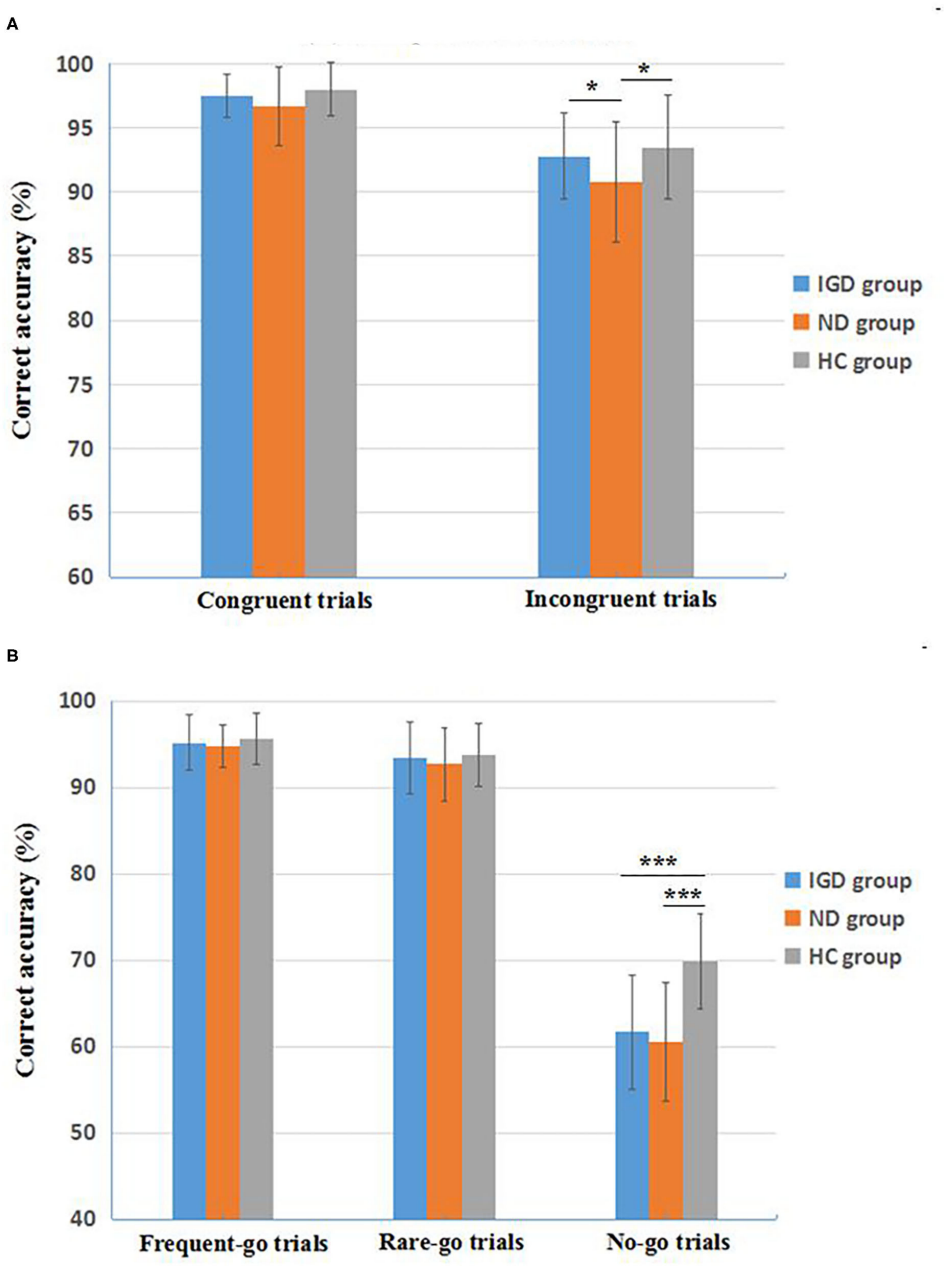
Inhibitory control performance on the Stroop Color-Word Task **(A)** and the revised Go/No Go Task **(B)** of the three groups. IGD, Internet Gaming Disorder; ND, Nicotine Dependence; HC, healthy controls. Data are presented as mean ± standard deviation (*M* ± *SD)*. *p < 0.05, ***p < 0.001.

The inhibitory control performance on the Stroop Color-Word Task and Go/No Go Task of the IGD, ND, and HC groups are showed in Table 3. On the Stroop task, the mANOVA models revealed that the group effects on correct accuracy were significant in the incongruent trials [*F*_(2, 162)_ = 6.351, *p* = 0.002, $\eta_{\text{p}}^{2}$ = 0.073] but not in the congruent trials [*F*_(2, 162)_ = 2.648, *p* = 0.076], and the group effects on response time were not significant in the congruent or incongruent trials [*F*_(2, 162)_ = 1.104, *p* = 0.334; *F*_(2, 162)_ = 0.682, *p* = 0.507, respectively]. Pairwise comparisons found that ND group had a lower correct accuracy in the incongruent trials compared with healthy controls (Cohen's *d* = 0.61, *p* = 0.001) and IGD group (Cohen's *d* = 0.49, *p* = 0.017), but IGD group did not differ from healthy controls (*p* = 0.250). Main effects of gender and interaction effects of group × gender were not significant on the correct accuracy and response time (*ps* > 0.05). See Figure 1A for a clear portrayal of the Stroop performance.

**Table 3:** Inhibitory control performance on the Stroop and Go/No Go tasks *(M* ± *SD)*.

**Variables**	**a. IGD (*n* = 58)**	**b. ND (*n* = 53)**	**c. HC (*n* = 57)**	***F***	***p***	***Post-hoc* test (*p* < 0.05)**
**Stroop color-word task**						
Correct accuracy in CC trials (%)	97.53 ± 1.69	96.72 ± 3.05	97.99 ± 2.09	2.780	0.067	-
Correct accuracy in IC trials (%)	92.80 ± 3.39	90.81 ± 4.66	93.50 ± 4.08	6.454^**^	0.002	a>b, c>b
Response time in CC trials (ms)	557.8 ± 65.6	568.9 ± 56.1	548.4 ± 56.9	1.617	0.202	-
Response time in IC trials (ms)	642.3 ± 94.8	664.0 ± 86.8	653.9 ± 92.6	0.781	0.460	-
**Go/No Go task**						
Correct accuracy in frequent-go trials (%)	95.20 ± 3.16	94.75 ± 2.44	95.67 ± 2.96	1.413	0.246	-
Correct accuracy in rare-go trials (%)	93.48 ± 4.18	92.71 ± 4.23	93.82 ± 3.67	1.074	0.344	-
Correct accuracy in no-go trials (%)	61.65 ± 6.60	60.55 ± 6.83	69.85 ± 5.47	36.372^***^	<0.001	c>a, c>b
Response time in frequent-go trials (ms)	165.0 ± 45.7	164.0 ± 55.2	175.1 ± 29.8	1.070	0.345	-
Response time in rare-go trials (ms)	200.1 ± 32.7	198.4 ± 44.0	209.0 ± 32.2	1.351	0.262	-

*IGD, Internet Gaming Disorder; ND, Nicotine Dependence; HC, Healthy Controls; CC, Congruent Condition; IC, Incongruent Condition*.

** *p < 0.01*,

*** *p < 0.001*.

On the Go/No Go task, the mANOVA models revealed significant group effects on correct accuracy in the no-go trials [*F*_(2, 162)_ = 38.160, *p* < 0.001, $\eta_{\text{p}}^{2}$ = 0.320], but not in the frequent-go trials [*F*_(2, 162)_ = 1.085, *p* = 0.350] or the rare-go trials [*F*_(2, 162)_ = 0.986, *p* = 0.375]. Group effects on response time were not significant in the frequent-go or rare-go trials [*F*_(2, 162)_ = 0.884, *p* = 0.415; *F*_(2, 162)_ = 0.939, *p* = 0.393, respectively). Pairwise comparisons found that both IGD and ND groups had a lower correct accuracy in the no-go trials than healthy controls (Cohen's *d* = 1.35, *p* < 0.001; Cohen's *d* = 1.50, *p* < 0.001, respectively), but the accuracy difference between IGD and ND groups was not significant (*p* = 0.332). Main effects of gender and interaction effects of group × gender were not significant on the Go/No Go accuracy and response time (*ps* > 0.05). See Figure 1B for a direct description of the Go/No Go performance.

### Correlations Between Gaming/Smoking Variables and Task Performance

Partial correlations were tested between Internet gaming variables (i.e., IAT score, years of regular gaming, daily gaming hours), nicotine use variables (i.e., FTND score, years of smoking, cigarettes per day) and task performance (i.e., BIS-11 scores, DDT and PDT scores, and inhibitory control scores), controlling for gender, age, ethnicity, and home locality. The data showed that most of the correlations were not significant between gaming/smoking variables with trait impulsivity, discounting degrees, and inhibitory control (*ps* > 0.05), except for that of the BIS-11 Non-planning Impulsiveness with FTND score, years of smoking, and cigarettes per day in the ND group (*r*_p_ = 0.267, *p* = 0.05; *r*_p_ = 0.269, *p* = 0.049; *r*_p_ = 0.278, *p* = 0.042, respectively). Please see more details for the partial correlations in the Supplementary Table 1.

## Discussion

The current study contrasted the characteristics of monetary reward discounting, inhibitory control, and trait impulsivity between Internet Gaming Disorder (IGD) and Nicotine Dependence (ND) individuals, with a well-matched sample of healthy controls as the reference group. To our best knowledge, this is the first study that has targeted a straightforward comparison between IGD and ND on reward processing and cognitive control aspects among young adults. Our data demonstrated that both the IGD and ND groups scored higher on the trait impulsivity (i.e., Motor, Attentional, and Non-planning Impulsiveness) and had higher degrees of delay discounting (i.e., poorer capability of delay gratification) than the healthy controls, while only the ND group showed a lower degree of probability discounting (i.e., lower risk aversion) than healthy controls. Moreover, IGD and ND groups displayed similar impaired inhibitory control on the revised Go/No Go task (i.e., a lower correct accuracy in No-Go trials) compared with healthy controls, but on the Stroop task only the ND subjects showed a lower correct accuracy in the incongruent trials than healthy controls. These findings suggested that IGD was linked to anomalous reward discounting and dysfunctional inhibitory control, comparable with one typical SUD category (i.e., ND) in this study.

In regard to trait impulsivity assessed with the BIS-11, plentiful studies have observed elevated scores among adolescents and young adults with IGD on the three dimensions (i.e., Motor, Attentional, and Non-planning Impulsiveness) (23, 31, 54, 89, 113), despite that some studies revealed no differences on these impulsiveness scores between treatment-seeking patients diagnosed with IGD and healthy controls (24). Our data were consistent with the results of most previous studies, revealing an increased level of trait impulsivity on the BIS model among individuals with IGD. Furthermore, our study detected similar elevated scores of the three dimensions in the Nicotine Dependence (ND) group, in line with the literature of trait impulsivity in cigarette smoking (114). These findings, together with our previous cross-sectional data of trait impulsivity in problematic Internet use and smoking behaviors, indicated that IGD showed a tendency of increased impulsivity traits comparable to ND (25). Interestingly, we further found that the BIS-11 scores were not significantly correlated with the severity of Internet gaming (Supplementary Table 1), but more serious nicotine use (e.g., years of smoking) was associated with a higher score on certain impulsivity trait (non-planning impulsiveness), indicating a possible toxic effect of ND on impulsivity. Particularly, nicotine (i.e., the primary component of cigarettes smoking and ND) is a specific agonist of nicotinic acetylcholine receptors (nAChRs), and chronic exposure to nicotine acts as a neuroteratogen by providing excessive cholinergic stimulation in the developing brain (115). Thus, the deleterious effects of ND are mostly connected with nicotine-induced overstimulation that causes overt neurotoxicity and the adaptive desensitization of the nAChRs that causes alterations in cholinergic transmission, which may produce derangements in final neuronal architecture such as the prefrontal cortex, resulting in less prefrontal inhibition and higher levels of impulsive trait (116).

On the monetary reward discounting tasks (i.e., DDT and PDT), our data showed that both IGD and ND groups had a higher delay-discounting degree (log-transformed *k* value) than the healthy controls, with a medium to large effect size (Cohen's *d* = 0.53–0.69), and no difference was found on the DDT between IGD and ND groups. These data were consistent with previous reports indicating higher degrees of delay discounting among adolescents and young adults with IGD (53–58), as well as among young heavy smokers and nicotine-dependent individuals (59–63). In this respect, an inability to delay gratification might be reflected both in IGD and ND subjects, and this similar tendency on discounting long-term rewards faster could play an important role in the development of these two disordered behaviors among the youths (24, 65). Furthermore, the partial correlations in our study did not find significant relationships between gaming/smoking severity with delay-discounting degrees (*k* values) in IGD and ND groups (Supplementary Table 1), probably suggesting that the poor delay gratification might not be aggravated by the severity of IGD or ND among these individuals. However, given the cross-sectional design of our study, whether the poor delay gratification is a predisposing factor for IGD or ND still needs more powerful longitudinal evidence.

With respect to probability discounting, IGD subjects did not differ from healthy controls on the probability-discounting degrees (log-transformed *h* values), indicating a normal risk aversion as expected in previous studies (53, 54), though inconsistent with some reports showing that IGD participants chose more probabilistic rewards than recreational Internet game users and controls (66–68). By comparison, the ND group had a lower degree of probability discounting than healthy controls with a medium effect size (Cohen's *d* = 0.55), suggesting a reduction in risk aversion or a greater risk-taking tendency (63, 117). However, as previous research pointed out, the floor effects of low probabilities in different studies might lead to inconsistent results (60, 69–71). Considering that our ND group merely scored lower than the controls on the Part A (i.e., $20 vs. $80), but neither on the Part B (i.e., $40 vs. $100) nor on the Part C (i.e., $40 vs. $60) of the PDT, it appears that the magnitudes of the risky and/or the constant monetary rewards might be also important in the choice of these addicted individuals (118), which calls for further investigations on this interesting topic that to what extend the probabilities and the magnitudes of monetary rewards may affect the degrees of probability discounting on the PDT.

Cognitive control or inhibitory control plays a crucial part in our goal-directed behaviors (48, 119), as well as in the uncontrolled addictive behaviors (120, 121). Impairments in cognitive control might affect the daily life, family relations, and occupational status of drug-dependent individuals, and are essential in the treatment and relapse of drug addiction (122). Based on the phenomenological and empirical evidence of decreased executive control in adolescents with IGD, some theoretical models of IGD, such as the tripartite neurocognitive model (46) and the Interaction of Person-Affect-Cognition-Execution model (I-PACE) (123), coincidentally underlined the key role of reduced cognitive control or inhibitory control in the development and maintenance of IGD. In the present study, we used the Stroop Color-Word Task and a revised Go/No Go task to test inhibitory control. The data on Stroop task revealed that the Nicotine Dependence (ND) subjects showed a lower correct accuracy than healthy controls in the incongruent trials (Cohen's *d* = 0.61), indicating a dysfunctional inhibitory control in heavy smokers (101), while the IGD subjects had similar performance on the Stroop as the healthy controls did, discordant with previous reports among adolescents showing that high school students with IGD made more errors in the incongruent trials than healthy controls, indicating impaired inhibitory control (85–90). Considering the obvious differences of the IGD samples in ours and other studies (i.e., young adult university students vs. adolescent high-school students), more attention should be paid to these contradictory findings in various samples of IGD. Especially, we noticed that although significantly differing from the healthy controls on the Stroop task, our ND subjects seemed not so “impaired,” with an average correct accuracy of 90.81% in the incongruent trials, in contrast to that of 92.80% (IGD group) and 93.50% (HC group). Thus, the task difficulty and complexity issues should be considered in future similar studies using the Stroop task.

More important findings in this study were from the revised Go/No Go task. Our data showed that the IGD and ND groups exhibited similar impaired performance of inhibitory control on this task (i.e., a lower correct accuracy in No-Go trials) than the healthy controls, with a large effect size (Cohen's *d* = 1.35, 1.50, respectively), yet the performance differences in frequent-Go and infrequent-Go trials among the IGD, ND and healthy control groups were not significant. Because of the potential confusions arising from the dual processing of attentional bias related to the novelty and response inhibition related to the no-go signals in traditional Go/No-Go paradigms (i.e., 25% No-Go trials vs. 75% Go trials), the present literature of the performance on the Go/No-Go task has been greatly inconsistent, with some studies reporting reduced inhibitory control functions in adolescents and young adults with IGD (31, 89), while others indicating intact inhibitory control in IGD subjects (86–88) and in heavy smokers (99, 100). Our current study firstly dissociated inhibitory control aspects from attentional bias among IGD and ND samples, using the newly modified and validated Go/No-Go task (89, 90) that contains 75% frequent-Go trials, 12.5% infrequent-Go trials, and 12.5% No-Go trials to directly detect response inhibition by comparing the infrequent-Go trials with No-Go trials. Our data clearly depicted that although the IGD and ND groups performed normally in both frequent-Go and infrequent-Go trials (the average correct accuracy >92%) similar to the healthy controls, these two disordered groups displayed apparent inhibitory impairments in the infrequent No-Go trials (an average correct accuracy of about 60%) compared with healthy controls (about 70% correct) (Figure 1B). Furthermore, the IGD individuals who has never used any addictive substance, still manifested a comparable impairment of inhibitory control on this task with the ND subjects, who probably had the concomitant intoxication consequences due to chronic nicotine use (116). These findings might indicate a basic pathology mechanism of cognitive control implicated in IGD as a potential addictive disorder similar to SUD (84). Nevertheless, we did not find a significant correlation between the inhibitory control impairments and the severity of smoking in the ND group, inconsistent with previous reports (96, 97). In light of the non-clinical samples of ND (i.e., university students) in our study, we speculated that a narrow distribution of smoking severity (e.g., an average score of 5.83 ± 1.03 on the FTND) might negatively affect the correlation results, and further studies with a larger ND sample are warranted to detect their accurate relationships.

There are several limitations that should be noted in the present study. Firstly, despite the fact that we mainly included the IGD and ND groups by person-to-person screening interview conducted by a psychiatrist and a clinical psychologist, according to the clinical criteria for IGD and ND, we also used some self-report scales (i.e., the IAT and the FTND) to evaluate the severity of IGD/ND, which might bring subjective biases, thus the results should be explained carefully. Secondly, our samples of IGD and ND primarily consisted of the young adult university students, which could not represent the whole population, so the findings should be further examined with other different samples (e.g., treatment-seeking populations of IGD and ND). Thirdly, we did not combine any neurophysiology measurement together with our behavioral tasks as did in previous studies [e.g., (68, 89, 95, 101)], thus our findings of deficient reward processing and inhibitory control in IGD and ND were short of powerful converged evidence on neurobiological correlates. Actually, the current literature suggests that functional and structural neural alterations in the fronto-striatal and fronto-cingulate regions are closely associated with IGD (81), and abnormal activities in the prefrontal areas (e.g., dorsolateral prefrontal cortex, anterior cingulate cortex, and orbitofrontal cortex/ventromedial prefrontal cortex) may account for the impaired cognitive control and decreased loss sensitivity in IGD and gambling disorder (124, 125) as well as for the craving and impulsivity in comorbid IGD and SUD (126). Thus, neurophysiology measurements should be better integrated with behavioral tasks in future. Additionally, although we contrasted IGD and ND individuals with well-matched healthy controls on multiple tasks, with a larger sample size relative to some previous studies [e.g., (23)], our case-control study design was cross-sectional in nature, therefore our results could not draw a causal conclusion, which should also be interpreted more discreetly.

Despite these limitations, our findings indicated for the first time that in a straightforward comparison, young adults with IGD and those with ND concurrently shared poorer capability of delay gratification as well as impaired inhibitory control, suggesting that IGD was linked to a neuropsychological pattern of anomalous reward discounting and dysfunctional inhibitory control, which was comparable to a typical SUD category (i.e., ND). This study thus might promote a better understanding of the pathogenesis of IGD as a potential addictive disorder similar to SUD. Furthermore, our first direct findings from a comparison between IGD and ND should be beneficial for potential clinical implications in the prevention and treatment of excessive Internet gaming and IGD, for instance, developing possible non-pharmacological therapeutic methods aimed at the restoration of inhibitory control or cognitive control functions (124) and/or reducing the high-level subjective representations of exciting activities or instant craving (127). In this respect, non-invasive neuromodulation methods, such as the repetitive transcranial magnetic stimulation (rTMS), have been proposed as an effective intervention to target cognitive dysfunctions in substance-related addictive disorders including tobacco, alcohol, and cocaine addiction (128). In view of the similar impaired inhibitory control detected in both IGD and ND in our study, future treatment of pathological Internet gaming might also be inspired to yield encouraging results by combining neuromodulation methods (e.g., rTMS) that could be applied on selected brain areas especially the left dorsolateral prefrontal cortex (DLPFC), which has been proved to probably improve the prefrontal top-down executive control and reduce drug craving and consumption in SUD, including ND (128, 129). However, there remains a big need for more accurate studies that can provide deeper insight into the core pathogenesis of IGD so as to advance this field, furnishing the foundation for developing an ideal model for practice in the prevention and treatment of IGD (130).

## Data Availability Statement

The raw data supporting the conclusions of this article will be made available by the authors, without undue reservation.

## Ethics Statement

The studies involving human participants were reviewed and approved by the Human Research Ethics Committee at the Guizhou Medical University. The patients/participants provided their written informed consent to participate in this study.

## Author Contributions

W-SY designed the study, wrote the protocols, directed the study, and wrote a first draft of the manuscript. R-TC, M-ML, and D-HZ performed the task assessments, data collection, and main data analysis. All of the authors contributed to the writing and have approved the final manuscript.

## Conflict of Interest

The authors declare that the research was conducted in the absence of any commercial or financial relationships that could be construed as a potential conflict of interest.
