# Supplementary material for: Monetary Reward Discounting, Inhibitory Control, and Trait Impulsivity in Young Adults With Internet Gaming Disorder and Nicotine Dependence
Source: Front Psychiatry. 2021 Jan 28;12:628933. doi: 10.3389/fpsyt.2021.628933 (PMC7876248; doi:10.3389/fpsyt.2021.628933)
Supplement: Supplementary file 1 [file Table_1.DOC]

**Supplementary Material** for this manuscript: Monetary Reward Discounting, [Inhibitory Control](http://www.baidu.com/link?url=-ShChg4ClUoHu2GihaunM9SHRq0-098vsZ13Jng52Z8YGfzRAuSuqA9n0-cxdakVtx3pD9a2HAmVtLcieBJ1gZzCcPcJyvWE9v2WCGWDwlOB0jhZ8uROhsLY1JPAUnJg), and Trait Impulsivity in Young Adults with Internet Gaming Disorder and Nicotine Dependence

**Table S1.** Partial correlations (*r*_p_) between gaming/smoking variables and task scores.

| **Variables** | **In IGD group (*n*=58)** | | | **In ND group (*n*=53)** | | |
| --- | --- | --- | --- | --- | --- | --- |
|  | IAT score | Years of gaming | Daily gaming hours | FTND score | Years of smoking | Cigarettes per day |
| **BIS-11** |  |  |  |  |  |  |
| Motor Impulsiveness | 0.186 | 0.215 | 0.175 | 0.205 | 0.117 | 0.173 |
| Attentional Impulsiveness | 0.147 | 0.135 | 0.160 | 0.028 | 0.107 | 0.158 |
| Non-planning Impulsiveness | 0.255 | 0.141 | 0.122 | 0.267* | 0.269* | 0.278* |
| **DDT** |  |  |  |  |  |  |
| *k* value (log-transformed) | -0.088 | -0.119 | -0.111 | 0.017 | 0.151 | 0.032 |
| **PDT** |  |  |  |  |  |  |
| Part A *h* value (log-transformed) | -0.081 | -0.096 | -0.070 | -0.043 | -0.036 | -0.007 |
| Part B *h* value (log-transformed) | 0.005 | -0.073 | -0.027 | -0.105 | -0.065 | -0.038 |
| Part C *h* value (log-transformed) | -0.150 | -0.154 | -0.072 | 0.046 | 0.051 | 0.116 |
| **Stroop Color-Word Task** |  |  |  |  |  |  |
| Correct accuracy in CC trials | -0.107 | -0.073 | -0.087 | -0.106 | 0.035 | -0.050 |
| Correct accuracy in IC trials | -0.072 | -0.095 | -0.053 | -0.139 | 0.013 | -0.166 |
| **Go/No Go Task** |  |  |  |  |  |  |
| Correct accuracy in frequent-go trials | 0.070 | 0.152 | 0.127 | 0.073 | -0.021 | 0.188 |
| Correct accuracy in rare-go trials | 0.187 | 0.172 | 0.159 | -0.015 | -0.121 | 0.063 |
| Correct accuracy in no-go trials | -0.008 | 0.050 | 0.095 | 0.105 | 0.050 | 0.036 |

Note. IGD=Internet Gaming Disorder, ND=Nicotine Dependence, IAT=Internet Addiction Test, FTND=Fagerström Test for Nicotine Dependence, BIS=Barratt Impulsiveness Scale, DDT=Delay-discounting Test, PDT=Probability Discounting Test, *k* represents the delay-discounting degree, *h* represents the probability-discounting degree, CC=Congruent Condition, IC=Incongruent Condition. **p*<0.05. Control variables: gender, age, ethnicity, and home locality.
